# Determination of Essential Oil Composition from *Osmanthus* *fragrans* Tea by GC-MS Combined with a Chemometric Resolution Method

**DOI:** 10.3390/molecules15053683

**Published:** 2010-05-19

**Authors:** Chun-Di Hu, Yi-Zeng Liang, Fang-Qiu Guo, Xiao-Ru Li, Wei-Ping Wang

**Affiliations:** 1 Pharmaceutical School of Xianning University, Xianning 437100, China; E-Mail: hcd918@163.com (C.D.H.); 2 Research Center of Modernization of Chinese Herbal Medicines, College of Chemistry and Chemical Engineering, Central South University, Changsha 410083, China; 3 Xinjiang Institute for Food and Drug Control, Urumqi 830002, China

**Keywords:** *Osmanthus fragrans* tea, essential oil, GC-MS, chemometric resolution method, temperature-programmed retention indices

## Abstract

The essential oils of the *Osmanthus fragrans* tea (OFT), *Osmanthus fragrans* (OF) and green tea (GT) had been extracted by steam distillation and analyzed by gas chromatography-mass spectrometry (GC-MS) with the help of heuristic evolving latent projections (HELP), an effective chemometric resolution method (CRM). The overlapping peak clusters were resolved into pure chromatograms and pure mass spectra with HELP. The qualitative analysis was performed by similarity searches in the National Institute of Standards and Technology (NIST) mass spectra database with the obtained pure mass spectrum of each component. Identification of some compounds was also assisted by comparison of temperature-programmed retention indices (PTRI) with authentic standards included in our own laboratory database under construction. The quantitative results were obtained by overall volume integration (OVI) method. A total of 67, 73 and 53 components in essential oils of the OFT, OF and GT were identified, accounting for 90.83%, 93.65% and 89.97% total contents of the essential oil of OFT, OF and GT, respectively.

## 1. Introduction

China is the homeland of tea, taking a leading position in the planting, producing and drinking of tea. The discovery and usage of tea has a history of four or five thousand years in China [1]. Tea has been reported to possess multiple functions such as prevention from cancer [2], hypotensive effects [3], antiviral properties [4], anti-oxidative properties [5], the inhibition of plaque formation [6], anti-allergic potential [7], blood glucose-lowering effects [8], and great benefits to health in general.

In order to increase the effectiveness or improve the taste of tea, some traditional Chinese medicines or flowers are often added to tea. Flavored teas are prepared by adding a range of fruits and flowers to the tea prior to final packaging. The added flowers are *Prumus mume*, *Paeonia suffruticosa*, *Jasminum* and *Osmanthus fragrans*, *etc.*, whose efficacy has been attributed to the volatile constituents. Very little is yet known about the qualitative and quantitative compositions of the volatiles of them, so the analysis of their constituents represents a very important step in the evaluation of efficacy and safety.

*Osmanthus fragrans* (OF), also known as Sweet Olive and Tea Olive, is a species of *Osmanthus* native to Asia, from the Himalaya east through southern China to southern Japan. OF has a strong fragrance, so OF is a precious raw material for food industry and spice industry. It is usually served as an additive in food, tea and other beverages. In Chinese, OF was infused with green tea (GT) to create a scented tea called *Osmanthus fragrans* tea (OFT).

For a better understanding of the physiological and pharmacological effects of tea, it is essential to scrutinize its chemical composition. There exist volatile and non-volatile components in tea. The essential oil is currently considered to be responsible for the smell and taste and part of the functions.

A great deal of works about tea volatiles have been reported [9,10,11]. But as far as we know, the volatile components in OFT have not yet been reported. In this paper, the identification of the volatile constituents of OFT is given, and a comparison and discussion with OF and GT are also made for the first time.

In general, the analysis of volatile components is usually conducted by using gas chromatography (GC) and gas chromatography-mass spectrometry (GC-MS) [12]. GC and GC-MS have greatly increased our knowledge of tea aroma. They are certainly useful and powerful tools in analysis of the essential oils of tea. In the case of GC, however, the identification of chromatographic peaks is typically just carried out by comparison of retention times with those of reference compounds. This means some of the components may not be identified due to the complexity of the matrix. On the other hand, retention times have no uniform criterions because they are affected by many factors. For GC-MS technique, more components are qualitatively and quantitatively analyzed, but the determination is performed only through the direct similarity searches in a MS database. There exist at least two serious problems for this approach [13]. First, the background cannot be accurately corrected. Second, as the boiling points of components analyzed are close to each other, there is a great probability that two or more components will co-elute, even when the chromatographic conditions are optimized. In such a case, is hard to identify the components by direct similarity search in a MS database alone, which can even result in wrong conclusions. Therefore, it is extremely necessary to resolve the overlapping peaks into a pure chromatogram by means of higher chromatographic separation capacities and chemometric resolution methods (CRM) [14]. Comprehensive two-dimensional gas chromatography (GC×GC) and the combination of a third mass spectrometric dimension to a GC×GC system are the most powerful analytical tool today for volatile and semi-volatile analysis [15,16]. In our work, we focus on using the GC-MS hyphenated technique to get two dimensional data and then on resolving the overlapping peaks into pure spectra and chromatograms with CRM. Up to now, various chemometric methods have been proposed to extract pure spectra from complex chromatograms, such as evolving factor analysis (EFA) [17], window factor analysis (WFA) [18], orthogonal projection resolution (OPR) [19], heuristic evolving latent projections (HELP) [20], evolving window orthogonal projections (EWOP) [21], sub-window factor analysis (SFA) [22], orthogonal projection approach (OPA) [23], iterative key set factor analysis (IKSFA) [24] and simplified Borgen method (SBM) [25].

The above mentioned methods can be roughly divided into iterative or non-iterative ones. Some papers [26] have been published comparing these methods. Although all these methods have been applied to solve some practical problems successfully and their limits have been investigated using simulated and/or experimental data, it is difficult to determine which method is the most suitable under any particular conditions. The HELP method, which was reported in 1992 by Kvalheim and Liang [20], has been extensively applied in the resolution of many complex mixtures. It makes full use of all chemical information obtained from the hyphenated analytical instrument and is proved to be a far better method than both EFA and OPA [27]. Unlike many other methods for curve resolution, the HELP method is fast, easy to use, and easy to understand. The HELP method thus possesses features that make it a promising approach for solving the complicated problems. Consequently, in this work, after getting two dimensional data using the GC-MS hyphenated technique we resolved the overlapping peaks into pure spectra and chromatograms with the HELP method.

Furthermore, temperature-programmed retention index *I^T^* was also employed for identification of the compounds. With the combination of retention index and mass spectra similarity search, the reliability of the qualitative analysis is highly improved.

## 2. Results and Discussion

### 2.1. Qualitative analysis

Richmond [28] indicated that *I^T^* can be highly reproducible on a given capillary column with a fixed stationary phase, if other working variables, such as carrier flow-rate, film thickness and linear temperature programming rate are standardized. For those substances whose mass spectra are similar to each other, the identification by direct mass spectral searching alone is difficult. However, they have different retention indexes, from which the qualitative results can be determined. Retention indexes and mass spectra are complementary to each other. Therefore, by making use of our *I^T^* database under construction, the results of identification of the compound will be more accurate. The combination of *I^T^* and the HELP method greatly improved the accuracy of identification of the chemical components in our essential oil samples.

A quasi-linear equation proposed by van Den Dool and Kratz [29] for temperature-programmed retention index was used to calculate *I^T^* in the present work:

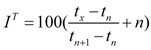
(1)
where *I^T^* is the temperature-programmed retention index of the interesting compound; t_n_, t_n+1_, and t_x_ are the retention times (in minute) of the two standard *n*-alkanes containing *n* and *n* + *1* carbons and the compound of interest, respectively.

**Figure 1:**
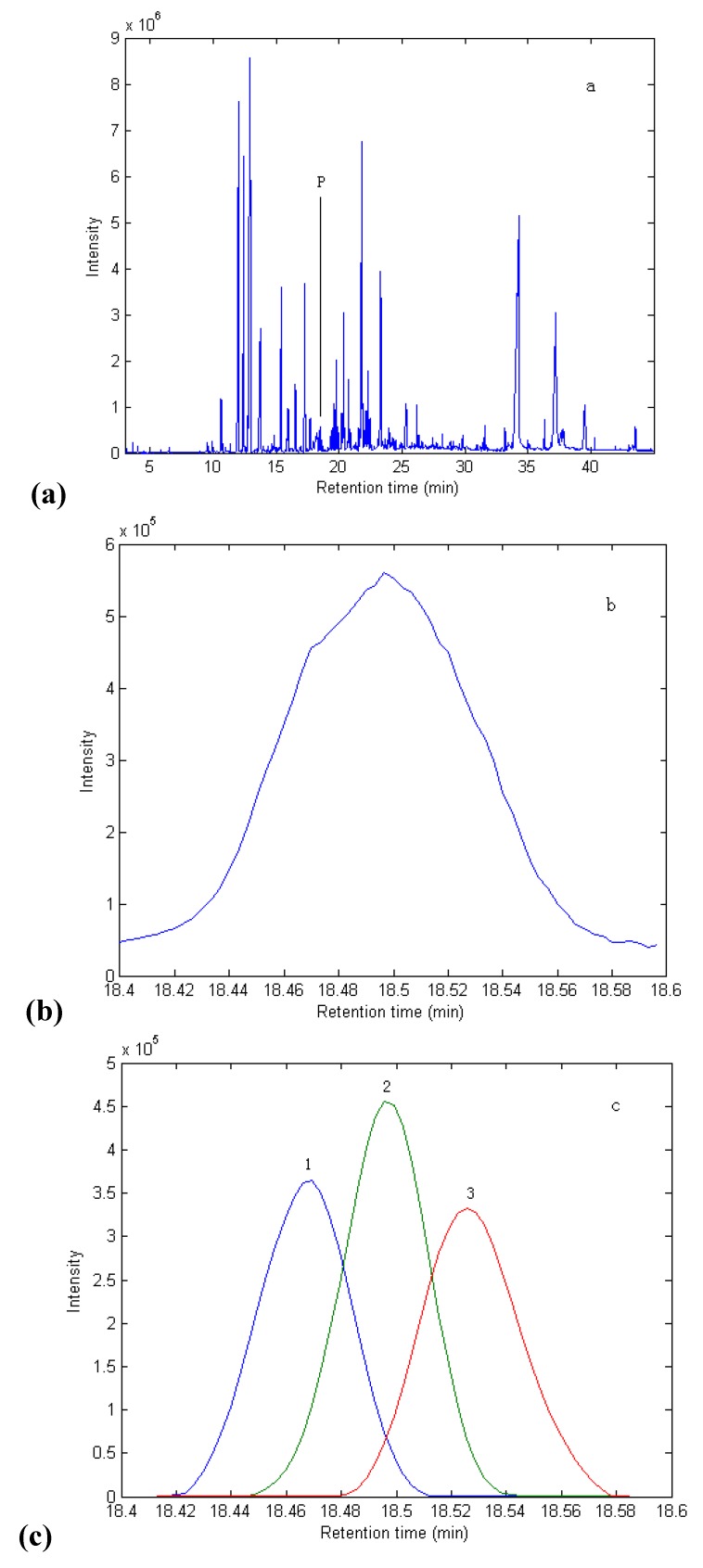
**(a)** TIC of essential oil of OFT. **(b)** TIC curve for overlapping peak P (within 18.4–18.6 min). **(c)** Resolved chromatograms for overlapping peak P containing components 1, 2 and 3.

The total ion chromatogram (TIC) of the essential oil of OFT is shown in Figure 1(a). As could be seen from Figure 1(a), there are a lot of peaks and their contents vary greatly. Although chromatographic separation was optimized, some of eluted components overlap with one another. If the NIST mass database were directly searched without further data processing for these overlapping peaks, incorrect identification of these compounds might be obtained. However, if the overlapping peaks were resolved into pure spectra and chromatograms with the HELP method, the qualitative analysis of components would be improved to a reliable extent. Here is the brief depiction of HELP method.

The data represented by **X**_m×n_ obtained from GC-MS is a bilinear matrix. According to the additive property of mass spectra, it can be expressed simply as follows:

**X**_m×n_ = **CS**^T^ + **E =** ∑**c**_i_**s**_i_^T^ + **E** ( i = 1, 2,…, N ) (2)

where **X**_m×n_ denotes an intensity matrix with N components of *m* chromatographic scan points at *n* atom mass units (amu). **C** and **S** are the pure chromatographic matrix and the pure spectral matrix, respectively. The superscript T represents the transpose of any matrix, in the present example **S**. **E** denotes the measurement noise.

Now peak cluster P (the retention time between 18.4 and 18.6 min) in Figure 1(a) is taken as an example to show the analytical procedure with HELP method. Figure 1(b) shows the amplified profile of peak cluster P, it seems to include one component. But the spectra from the start point to end point vary, which means it is not the same component. Direct search in the NIST mass database gives 5-methylene-4,5,6,6a-tetrahydro-3ah-pentalen-1-one for the middle of the peak cluster P with the similarity index 81%. However, for the front and the back of the middle in the same peak cluster P one can obtain different compounds with lower similarity indexes, so the conclusion can be drawn that peak cluster P contains overlapping peaks and it is necessary to resolve such overlapping peaks.

First of all, background, baseline shift and severe heteroscedastic noise in the raw dimensional data were pretreated by the corresponding methods [20]. Then the rank map was obtained with fixed size moving window evolving factor analysis (FSMWEFA) [20]. The rank map showed there were three components in peak cluster P. Three purified spectra were obtained, and after searching in the NIST database, they were identified as 1-(1,1-dimethylethyl)-4-ethylbenzene, 2,5,5,8a-tetramethyl-6,7,8,8a-tetrahydro-5H-chromen-8-ol and 2-methoxy-4-vinylphenol, with match factors of 91%, 93% and 94%, respectively. The corresponding obtained pure chromatographic profiles are shown in Figure 1(c).

With the same analytical procedure used for peak cluster P, the other clusters in the TIC can be analyzed one by one by the HELP method. The compounds resolved with HELP method are 15, which are listed in Table 1. A total of 63, 69 and 51 components were identified in essential oils of the OFT, OF and GT using similarity searches directly, while the numbers were extended to 67, 73 and 53 components using chemometric techniques. At the same time, the retention indexes were also used for the identification of the compounds along with the mass similarity search, especially to those with similar mass spectra. The retention index data of each component were also listed in Table 1.

**Table 1:** Compositions of the essential oil from OFT, OF and GT.

*I^T^*	Compound / formula	R. C.^a^ / %		
		**OFT**	**OF**	**GT**
782	Hexanal / C_6_H_12_O	0.05	-	- ^b^
858	Heptanal / C_7_H_14_O	-	-	0.1
877	2,6-Dimethyl-2,5-heptadiene / C_9_H_16_	-	-	0.13
963	2,2,6-Trimethyl-6-vinyltetrahydropyran / C_10_H_18_O	-	0.05	-
982	3-Hexadecyne / C_16_H_30_	0.13	-	-
984	β-Myrcene / C_10_H_16_	-	0.14	-
993	3-Dodecyne / C_12_H_22_	0.12	0.21	-
1018	Ethylhexanol / C_8_H_18_O	1.21	0.32	-
1020	4-Ethyl-1-octyn-3-ol / C_10_H_18_O	-	0.41	-
1021	D-Limonene / C_10_H_16_	0.11	0.19	-
1028	trans-β-Ocimene / C_10_H_16_	-	0.06	-
1039	cis-β-Ocimene / C_10_H_16_	0.1	0.11	-
1057	cis-Linalool oxide / C_10_H_18_O_2_	-	0.12	1.35
1061	1,2-Epoxylinalool / C_10_H_18_O_2_	7.17	15.32	0.76
1074	5-Ethenyltetrahydro-α,α,5-trimethyl-2-furanmethanol / C_10_H_18_O_2_	4.39	10.89	-
1079	(+)-4-Carene / C_10_H_16_	0.07	-	-
1084	Nonanal / C_9_H_18_O	0.55	0.5	0.39
1090	β-Linalool / C_10_H_18_O	14.47	11	11.12
1114	4-Cyclopentylidene-2-butanone / C_9_H_14_O	2.19	1.74	-
1119	3-Nonen-2-one / C_9_H_16_O	1.6	1.1	-
1140	Isoneroloxide / C_10_H_16_O	-	0.11	-
1149	Epoxylinalol / C_10_H_18_O_2_	0.26	0.1	-
1164	Limonene epoxide / C_10_H_16_O	-	0.07	-
1172	α-Terpineol / C_10_H_18_O	3.02	2.16	3.16
1182	Solanone / C_13_H_22_O	-	-	0.11
1189	Carvomenthenal / C_10_H_16_O	0.58	-	-
1191	1-p-Menthen-9-al / C_10_H_16_O	-	1.06	-
1193	8-Methyl-tricyclo[3.3.0.0(2,8)]octan-3-one / C_9_H_12_O	0.52	0.85 ^c^	-
1201	6-Ethyl-2-methyl-decane / C_13_H_18_	-	0.33 ^c^	0.12
1210	1,4-Diisopropyl-2-methylbenzene / C_13_H_20_	-	0.28 ^c^	-
1213	Nerol / C_10_H_18_O	1.08	0.63	1.27
1238	trans-Geraniol / C_10_H_18_O	3.26	1.89	7.02
1242	2,5,8-Trimethyl-1,2,3,4-tetrahydronaphthalene / C_13_H_18_	-	0.32	-
1245	Megastegma-7,9,13-triene / C_13_H_20_	-	0.05	-
1254	2-Cyclopropylidene-1,7,7-trimethyl-bicyclo[2.2.1]heptane / C_13_H_20_	0.52	0.8	0.16
1261	Undecanoic acid / C_11_H_22_O_2_	-	-	0.78
1263	6-Ethyl-7-hydroxy-4-octen-3-one / C_10_H_18_O_2_	0.09	-	-
1272	Nonanoic acid / C_9_H_18_O_2_	0.16	-	-
1280	1-(1,1-Dimethylethyl)-4-ethylbenzene / C_12_H_18_	0.2 ^c^	0.05	-
1281	2,5,5,8a-Tetramethyl-6,7,8,8a-tetrahydro-5H-chromen-8-ol / C_13_H_20_O_2_	0.29 ^c^	0.46	-
1282	2-Methoxy-4-vinylphenol / C_9_H_10_O_2_	0.18 ^c^	-	0.65
1286	Z,Z,Z-1,4,6,9-Nonadecatetraene / C_19_H_32_	0.14 ^c^	-	-
1287	Z,Z,Z-4,6,9-Nonadecatriene / C_19_H_34_	0.06 ^c^	0.24	-
1288	2,6,10,10-Tetramethyl-1-oxa-spiro[4.5]dec-6-ene / C_13_H_22_O	0.09 ^c^	-	-
1293	(1,4-Dimethylpent-2enyl)benzene / C_13_H_18_	-	0.06	-
1312	4,8-Decadienoic acid, 2-acetyl-2,5,9-trimethyl-, ethyl ester , (E) / C_17_H_28_O_3_	0.17	-	-
1315	Megastigma-4,6(E),8(E)-triene / C_13_H_20_	0.25	0.59	-
1327	2-Methoxy-3-(2-propenyl)-phenol / C_10_H_12_O_2_	-	-	0.76
1329	Megastigma-4,6(Z),8(E)-triene / C_13_H_20_	1.23	2.37 ^c^	-
1336	p-tert-Butylbenzyl alcohol / C_11_H_16_O	0.46	0.17 ^c^	-
1337	2-(2-Butenyl)-1,3,5-trimethyl-benzene / C_13_H_18_	-	0.16 ^c^	-
1340	Neric acid / C_10_H_16_O_2_	-	-	0.77
1343	Megastigma-4,6(E),8(Z)-triene / C_13_H_20_	0.51	1.05	-
1351	Megastigma-4,6(Z),8(E)-triene / C_13_H_20_	2.22	4.52	-
1354	2-Acetonylcyclohexanone / C_9_H_14_O_2_	0.27	-	-
1356	Decanoic acid / C_10_H_20_O_2_	-	-	0.2
1358	Eugenol / C_10_H_12_O_2_	0.1	-	-
1366	α-Ionol / C_13_H_22_O	1.12	1.37	-
1372	8-Cedren-13-ol / C_15_H_24_O	0.36	0.48	-
1389	Cubenol / C_16_H_26_O	-	0.08	-
1397	4-(2,6,6-Trimethylcyclohexa-1,3-dienyl)butan-2-one / C_13_H_20_O	0.37	0.77	0.15 ^c^
1403	α-Irone / C_14_H_22_O	-	-	0.13 ^c^
1405	4-(2,6,6-Trimethyl-1-cyclohexen-1-yl)-3-buten-2-ol / C_13_H_22_O	7.58	-	0.47 ^c^
1406	Cedrene / C_15_H_24_	0.74	0.61	-
1407	β-Ionol / C_13_H_22_O	-	5.73	-
1419	Dihydro-β-ionone / C_13_H_22_O	-	0.52	-
1425	γ-n-Heptylbutyrolactone / C_11_H_20_O_2_	1.49	-	-
1427	δ-Undecalactone / C_11_H_20_O_2_	-	1.26	-
1429	Geranyl acetone / C_13_H_22_O	-	-	0.21
1435	α-Guaiene / C_15_H_24_	-	0.39	-
1458	2,3-Epoxy-β-ionone / C_13_H_20_O_2_	-	-	0.19
1464	β-Ionone / C_13_H_20_O	3.37	2.64	0.62
1491	2,6-Bis(tert-butyl)phenol / C_14_H_22_O	0.29	-	1.62
1496	α-Farnesene / C_15_H_24_	-	0.69	0.25
1503	Plinol D / C_10_H_18_O	0.23	-	0.24
1512	9-n-Octyl-8-heptadecene / C_25_H_50_	0.14	0.12	0.21
1525	2-Methyl-4-(1-methylethyl)-2-cyclohexenone / C_10_H_16_O	-	-	0.25
1548	Nerolidyl acetate / C_17_H_28_O_2_	0.63	-	4.7
1575	Farnesene epoxide / C_15_H_24_O	-	-	0.64
1584	8-β-H-Cedran-8-ol / C_15_H_26_O	0.64	0.36	0.39
1591	Megastigmatrienone / C_13_H_28_O	0.16	0.11	-
1622	1β-Cadin-4-en-10-ol / C_15_H_26_O	-	-	0.14
1639	Patchouli alcohol / C_15_H_26_O	0.18	1.19	0.15
1679	3-Acetyl-4-hydroxy-6-methyl-2H-pyran-2-one / C_8_H_8_O_4_	-	2.65	-
1673	2-Hydroxy-6-methyl-3-(1-methylethyl)-2-cyclohexen-1-one / C_10_H_16_O_2_	0.2	-	-
1699	Nerolidol / C_15_H_26_O	-	-	0.28
1700	n-Heptadecan / C_17_H_36_	0.08	0.14	-
1712	2,6,10,14-Tetramethylpentadecane / C_19_H_40_	-	0.13	-
1719	8-Hexyl-pentadecane / C_21_H_44_	-	-	0.14
1797	Pentadecanal / C_15_H_30_O	-	0.09	-
1825	Butyl octyl phthalate / C_20_H_30_O_4_	0.16	0.1	0.34
1830	Hexahydrofarnesyl acetone / C_18_H_36_O	0.31	0.36	0.68
1838	3,7,11,15-Tetramethyl-2-hexadecen-1-ol / C_20_H_40_O	-	-	1.5
1879	1,2-Epoxyoctadecane / C_18_H_36_O	-	-	0.52
1893	Farnesyl acetone / C_18_H_30_O	-	-	0.09
1899	Octadecanal / C_18_H_36_O	-	0.08	-
1910	Methylhexadecanoate / C_17_H_30_O_2_	0.28	0.35	0.5
1916	Diisobutylphthalate / C_16_H_22_O_4_	-	-	0.36
1940	Isophytol / C_20_H_40_O	-	-	1.28
1961	Myristic acid / C_14_H_28_O_2_	1.36	4.24	0.76
1963	Hexadecanoic acid / C_16_H_32_O_2_	10.95	2.82	30.2
1967	Eicosanoic acid / C_20_H_40_O_2_	4.65	1.64	1.31
2002	Octadecanal / C_18_H_36_O	0.11	-	-
2003	1,2-Epoxyoctadecane / C_18_H_36_O	-	0.26	-
2072	8,11-Octadecadienoic acid, methyl ester / C_19_H_34_O_2_	0.14	0.16	0.32
2076	Methyl linolenate / C_19_H_32_O_2_	0.38	0.44	0.59
2100	Heneicosane / C_21_H_44_	-	0.1	7.36
2109	6-Pentadecen-1-ol / C_15_H_30_O	-	-	2.27
2112	8,11,14-Eicosatrienoic acid / C_20_H_34_O_2_	-	-	1.66
2120	2-cis,cis-9,12-Octadecadienyloxyethanol / C_20_H_38_O_2_	2.02	-	-
2125	11,14,17-Eicosatrienoic acid, methyl ester / C_21_H_36_O_2_	2.48	2.35	-
2148	9,9-Dimethoxybicyclo[3.3.1]nona-2,4-dione /C_11_H_16_O_4_	0.16	-	-
2200	n-Docosane / C_22_H_46_	1.95	-	0.23
2300	n-Tricosane / C_23_H_48_	0.16	0.24	-
2400	n-Tetracosane / C_24_H_50_	-	0.05	-
2472	Pentacosanol / C_25_H_52_O	-	0.11	-
2500	n-Pentacosane / C_25_H_52_	0.32	0.54	0.37
total		90.83	93.65	89.97

^a^ relative content; ^b^ not found; ^c^ These compounds were resolved with the HELP method.

### 2.2. Quantitative analysis

The overall volume integration method [13] was employed in this work on all the resolved chromatogram peaks in order to obtain the quantitative results of each component. The qualitative constituents of OFT, OF and GT account for 90.83%, 93.65% and 89.97% of the total essential oil contents, respectively. The quantitative results were also listed in Table 1.

### 2.3. Comparison of the volatile constituents from OFT, OF and GT

From Table 1, it can be known that, the main components from the essential oil of OF are 1,2-epoxylinalool, β-linalool, 5-ethenyltetrahydro-α,α,5-trimethyl-2-furanmethanol and β-ionol, accounting for 15.32%, 11%, 10.89%, and 5.73%, respectively. The main components from the essential oil of GT are hexadecanoic acid, heneicosane, *trans*-geraniol and nerolidyl acetate, accounting for 30.2%, 7.36%, 7.02% and 4.7%, respectively. The main components from the essential oil of OFT are β-linalool, hexadecanoic acid, and 4-(2,6,6-trimethyl-1-cyclohexen-1-yl)-3-buten-2-ol and 1,2-epoxylinalool, accounting for 14.47%, 10.95%, 7.58% and 7.17%, respectively. Apparently, there is significant difference in constituents and contents, which could be the main reason of difference of effectiveness and taste between GT and OFT. Furthermore, the constituents of OFT essential oil are not the simple additive result of GT and OF, the reason is mainly because of the some chemical reaction during the mix processing. Further research on these changes with regard to the pharmacological effects of OFT and GT is needed.

## 3. Experimental

### 3.1. Plant materials and n-alkane standard solution

Flowers of OF and GT from the Xianning producing areas (Hubei Province, China) were purchased at a store. The OFT was made of OF and GT with the ratio 1:1. *n*-Alkane standard solutions of C_8_-C_20_ (mixture No. 04070) and C_21_-C_40_ (mixture No. 04071) were purchased from Fluka Chemical Corporation.

### 3.2. Extraction of essential oil

Essential oils were extracted by water distillation for 5 h from dried materials, using a set of standard apparatus, according to the procedure described in the Chinese Pharmacopoeia [30]. The essential oils obtained were stored in the refrigerator prior to analysis. The yields of the essential oils of OFT, OF, GT were 0.16%, 0.15 and 0.13 (*v/w*), respectively.

### 3.3. GC-MS analysis

GC-MS analysis were performed on a QP2010 instrument equipped with an OV-1 capillary column (30 m × 0.25 mm, 0.25 μm). The oven temperature program initiated at 50 ºC, held for 3 min, then raised at 5 ºC/min to 250 ºC, held for 2 min. Other operation conditions were as follows: injector temperature, 250 ºC; carrier gas, He (99.999 %), adjusted to a column velocity of flow 1.0 mL/min; splitting ratio 10:1; interface temperature, 250 ºC; standard electronic impact (EI) MS source temperature, 200 ºC; mass scan range, 35–500 amu; scan velocity, 5 scans/s.

## 4. Conclusions

The essential oils of OFT, OF and GT had been analyzed for the first time by GC-MS combined with a chemometric resolution method. In total, 67, 73 and 53 components in essential oils of the OFT, OF and GT were respectively identified, accounting for 90.83%, 93.65% and 89.97% total contents of the essential oil of OFT, OF and GT, respectively. The major compounds from OFT, OF and GT were -linalool, 1,2-epoxylinalool and hexadecanoic acid, respectively.
